# Nuclear Smooth Muscle α-actin in Vascular Smooth Muscle Cell Differentiation

**DOI:** 10.21203/rs.3.rs-1623114/v1

**Published:** 2023-02-28

**Authors:** Callie S. Kwartler, Albert J. Pedroza, Anita Kaw, Pujun Guan, Shuangtao Ma, Xue-yan Duan, Caroline Kernell, Charis Wang, Jose Emiliano Esparza Pinelo, Mikayla S. Borthwick, Jiyuan Chen, Yuan Zhong, Sanjay Sinha, Xuetong Shen, Michael P. Fischbein, Dianna M. Milewicz

**Affiliations:** 1 Division of Medical Genetics, Department of Internal Medicine, McGovern Medical School, The University of Texas Health Science Center at Houston, Houston, TX 77030; 2 Department of Cardiothoracic Surgery, Stanford University, Stanford, CA 94305; 3 Current address: Department Medicine, Michigan State University, East Lansing, MI 48824; 4 Department of Epigenetics and Molecular Carcinogenesis, The University of Texas MD Anderson Cancer Center, Smithville, TX 78957; 5 Wellcome-MRC Cambridge Stem Cell Institute, University of Cambridge, Cambridge, United Kingdom; 6 Institute of Cancer Research, Shenzhen Bay Laboratory, Shenzhen, China

## Abstract

Missense variants throughout *ACTA2*, encoding smooth muscle α-actin (αSMA), predispose to adult onset thoracic aortic disease, but variants disrupting arginine 179 (R179) lead to Smooth Muscle Dysfunction Syndrome (SMDS) characterized by childhood-onset diverse vascular diseases. Our data indicate that αSMA localizes to the nucleus in wildtype (WT) smooth muscle cells (SMCs), enriches in the nucleus with SMC differentiation, and associates with chromatin remodeling complexes and SMC contractile gene promotors, and the *ACTA2* p.R179 variant decreases nuclear localization of αSMA. SMCs explanted from a SMC-specific conditional knockin mouse model, *Acta2^SMC-R179/+^*, are less differentiated than WT SMCs, both *in vitro* and *in vivo*, and have global changes in chromatin accessibility. Induced pluripotent stem cells from patients with *ACTA2* p.R179 variants fail to fully differentiate from neural crest cells to SMCs, and single cell transcriptomic analyses of an *ACTA2* p.R179H patient’s aortic tissue shows increased SMC plasticity. Thus, nuclear αSMA participates in SMC differentiation and loss of this nuclear activity occurs with *ACTA2* p.R179 pathogenic variants.

## Introduction

Heterozygous missense mutations in *ACTA2*, the gene encoding smooth muscle specific alpha-actin (αSMA), cause a diverse spectrum of vascular diseases, including thoracic aortic disease, premature coronary artery disease, and moyamoya-like cerebrovascular disease.^1, 2^ All identified *ACTA2* pathogenic variants cause thoracic aortic disease, likely due to the loss of smooth muscle cell (SMC) contraction, resulting in compensatory signaling by SMCs to correct for deficient homeostatic force generation.^3^ Only a subset of missense pathogenic variants in *ACTA2* are associated with non-thoracic aortic vascular diseases, and clinical data confirm that specific *ACTA2* missense variants are associated with a risk for either early onset coronary artery disease or moyamoya-like cerebrovascular disease.^1^ These significant genotype to phenotype correlations suggest *ACTA2* pathogenic variants alter αSMA by distinct mechanisms that have disparate effects on SMC phenotype.

*De novo* pathogenic variants that disrupt arginine 179 (R179), lead to a severe, childhood-onset syndrome termed Smooth Muscle Dysfunction Syndrome (SMDS).^4, 5^ SMDS patients have childhood onset thoracic aortic disease, moyamoya-like cerebrovascular disease, and pulmonary hypertension, along with patent ductus arteriosus and complications in the lungs, liver, gut, bladder, and eye.^4, 5^ Importantly, the clinical phenotype is similar no matter which amino acid substitution occurs, e.g. p.R179C vs. p.R179H. The moyamoya-like cerebrovascular disease in SMDS patients is characterized by occlusive lesions bilaterally in the distal internal carotid arteries and their branches without compensatory collateral vessel formation typical for classic moyamoya disease. These patients also have straightening of cerebral arteries and periventricular hyperdense lesions, suggestive of small vessel disease.^6, 7^ Histologic examination of cerebral vessels from an *ACTA2* p.R179H patient shows thickened medial layers and intimal lesions containing cells that stain positively for an SMC marker without signs of inflammation or lipids.^8^ The neointimal SMC accumulation in the cerebrovascular arteries suggests *ACTA2* p.R179 alterations increase SMC migration and proliferation.

Ubiquitously expressed β-actin is best known for its cytoskeletal roles in cell motility, but it also has defined functions in the nucleus. Nuclear β-actin associates with all three RNA polymerases and multiple ATP-dependent chromatin remodeling complexes, and polymerizes to promote nuclear structural integrity.^9^ Nuclear β-actin is a critical determinant of cell fate during mammalian development, and loss of nuclear β-actin prevents neuronal differentiation.^10, 11^ Our data preliminarily suggested that αSMA also enters the nucleus in SMCs,^12^ and in this study, we go on to confirm that αSMA accumulates in the nucleus during SMC differentiation, where it associates with the INO80 and BAF chromatin remodeling complexes and with the promoters of SMC marker genes. Ectopic increases in nuclear localization of αSMA promote expression of SMC differentiation markers. Furthermore, heterozygous *ACTA2* p.R179 variants reduce the nuclear localization of αSMA. These mutant SMCs have decreased levels of SMC differentiation markers, retain expression of pluripotency genes, and have global alterations in chromatin accessibility when compared to WT SMCs in multiple model systems. Based on the data presented here, we propose that nuclear αSMA is required for cell fate specification of vascular SMCs, and the absence of nuclear αSMA in patients with *ACTA2* R179 mutations leads to global defects in SMC differentiation.

## Results

### αSMA localizes to the nucleus of SMCs

We sought to confirm nuclear localization of αSMA in SMCs. WT SMC protein lysates were fractionated into nuclear and cytosolic fractions, and immunoblot analyses confirm presence of αSMA in both the cytosolic and nuclear fractions, both at baseline and after 48 hours of treatment with transforming growth factor β1 (TGFβ1) or 24 hours of treatment with platelet derived growth factor BB (PDGF-BB). Exposure to TGFβ1 increases expression and protein levels of SMC differentiation markers, including βSMA, and both cytosolic and nuclear levels of αSMA increase with TGFβ1 exposure (Fig. 1A, Supplemental Fig. IA). Exposure to PDGF-BB decreases expression and protein levels of SMC differentiation markers, but does not affect levels of αSMA in the nucleus (Fig. 1A, Supplemental Fig. IA). Fractionated lysates were also separated on a 2-dimensional gel system with isoelectric focusing prior to SDS-PAGE analysis, allowing separation of α-and β-actin in SMCs. The blots show both β-actin and αSMA localize to both the cytosol and nucleus at baseline and following 48 hours of TGFβ1 treatment (Fig. 1B, Supplemental Fig. IB).

Polymerized cytosolic αSMA filaments connect to the nucleus via the LINC complex, so cytosolic αSMA could potentially contaminate the nuclear fraction. Thus, cells were pre-treated with latrunculin A, which rapidly depolymerizes actin, one hour prior to harvesting. Neither nuclear nor cytosolic αSMA levels were affected by latrunculin treatment, nor was nuclear accumulation of β-actin (Fig. 1C, Supplemental Fig. IC).

SMCs were immunostained with an antibody against αSMA and imaged with structured illumination microscopy (SIM). Staining of αSMA in the nucleus overlaps with the nuclear DAPI stain, and co-staining for αSMA and the heterochromatin marker, HP1, shows a negative correlation between HP1 and αSMA staining, suggesting that αSMA localizes to open chromatin (Fig. 1D,F). Quantitation of αSMA fluorescent intensity within the nucleus shows that treatment with either TGFβ1 or PDGF-BB increases levels of nuclear αSMA (Fig. 1D,E). β-actin nuclear intensity does not change with growth factor treatment (Supplemental Fig. II). TGFβ1 treatment increases the negative correlation of αSMA with HP1, suggesting increased αSMC binding to open chromatin with increased differentiation, while de-differentiation associated PDGF-BB treatment decreases it (Fig. 1D,E). These results support that αSMA intranuclear localization changes with growth factor treatments that alter the phenotype of SMCs.

### αSMA enriches in the nucleus as SMCs differentiate

Based on the data that TGFβ-driven differentiation of SMCs increases αSMA localization to open chromatin, we assessed the nuclear αSMA during the initial specification of SMCs during development, using an *in vitro* model of differentiation from induced pluripotent stem cell (iPSC) to neuro-ectodermal progenitor cells (NEPC) to SMC.^13^ Cell lysates were harvested at the NEPC stage (day 0) and every four days during the 12-days of exposure to TGFβ1 and platelet-derived growth factor (PDGF)-BB used to drive NEPC to SMC differentiation, and then separated into nuclear and cytosolic fractions. We found that αSMA is in the nuclear but not cytosolic fraction in NEPCs at day 0, and as αSMA levels increase with SMC differentiation, the αSMA in the nuclear fraction increases earlier than cytosolic levels of αSMA (Fig. 1G, Supplemental Fig. ID). In contrast β-actin protein levels do not change dramatically over the 12 day time course, with low levels of nuclear β-actin in NEPCs that increase by day 4 and remain stable through day 12 (Fig. 1G, Supplemental Fig. ID). Exponential increases in the expression of SMC differentiation genes occur between day 0 and day 4 and expression remains elevated through day 12 (Fig. 1H). These data support that increased αSMA localization to the nucleus occurs with SMC differentiation.

### Nuclear αSMA associates with the INO80 and BAF chromatin remodeling complexes and binds to the promoters of SMC differentiation marker genes

Nuclear β-actin is a well-established member of multiple chromatin remodeling complexes, so co-immunoprecipitation analyses were performed to identify whether αSMA also associates with these complexes. Lysates of SMCs were immunoprecipitated with an αSMA antibody and immunoblot analyses of the pulldowns showed both the INO80 chromatin remodeling complex including YY1, INO80, and TIP49 and the BAF chromatin remodeling complex including BAF170, BAF57, BRG1, and BAF155 (Fig. 2A). Reciprocal pulldowns using an antibody directed against the INO80 complex component INO80 and an antibody directed against the BAF complex component BRG1 confirms that both αSMA and β-actin are found associated with these complexes (Fig. 2B,C). Association of both αSMA and β-actin with the INO80 complex but not the BAF complex increase with TGFβ1 treatment.

Nuclear αSMA potentially associates with the CArG box elements that are crucial for expression of SMC differentiation markers;^14^ thus, chromatin immunoprecipitation (ChIP) was performed using antibodies directed against either αSMA or β-actin in SMCs. Following immunoprecipitation, quantitative RT-PCR (qPCR) was performed using primers for the CArG box regions of SMC differentiation makers *Cnn1, Myh11*, and *Tagln*. All four promoter regions amplified in the immunoprecipitated DNA from both αSMA and β-actin pulldowns. After 48 hours of TGFβ1 driven differentiation, increased promoter amplification was found with αSMA pulldown but not β-actin, indicating an enrichment of αSMA at the promoters of SMC contractile genes with differentiation (Fig. 2D). By contrast, no significant changes in binding were found on the promoter of *Actb*.

To assess whether αSMA co-occurs with chromatin remodeling complexes on the SMC contractile gene promoters, a sequential ChIP experiment was performed. Pulldown with αSMA antibody followed by INO80 antibody revealed co-occupancy of the two proteins on the promoters of SMC-specific genes that could be confirmed by reciprocal pulldown using INO80 antibody followed by αSMA antibody (Figure 2E, single pulldown controls in Supplemental Figure III). The *Actb* promoter was negative for reciprocally confirmed co-occupancy. By contrast, BRG1 and αSMA did not show co-occupancy as sequential pulldowns did not reveal any enrichment over the IgG control.

### Forced nuclear localization of αSMA enhances levels of SMC contractile proteins

To further assess a role for nuclear αSMA in enhancing the expression of SMC differentiation markers, αSMA and β-actin were tagged with both a nuclear localization sequence (NLS) and a Flag epitope tag at the N-terminus, and then cloned into a lentiviral vector. These vectors were used to infect immortalized mouse SMCs, and fractionated lysates along with immunofluorescent staining showed enrichment of the Flag-tagged overexpressed protein in the nuclear lysates (Fig. 2F, Supplemental Fig. IVA). αSMA-NLS infection but not β-actin-NLS infection increases protein levels of the SMC specific proteins calponin, SM22α, and SMMHC (Fig. 2F, Supplemental Fig. IVB). To confirm the functional impact of αSMA-NLS, the cells were subjected to a collagen contraction assay. αSMA-NLS infection increased cellular contractility compared with β-actin-NLS or empty vector (EV) infection (Figure 2G).

The vectors were also transfected into 293T cells: 24 hours after transfection in the αSMA-NLS transfected cells there is significantly increased expression of *Myh11* and *Cnn1*, and by 48 hours there are increased levels of calponin protein; no such increases were identified in the β-actin-NLS infected cells (Supplemental Fig. VA-C). Similar results are obtained with transient transfection of HeLa cells, with only αSMA-NLS reproducibly increasing expression of *Myh11* and *Cnn1* (Supplemental Fig. VD-F). Importantly, HeLa and 293T cells lack myocardin and other machinery typically necessary for SMC marker gene expression, which may explain the relatively modest increases in levels seen with our NLS construct. Nonetheless, taken together, this data supports that nuclear αSMA drives expression of SMC markers in multiple cell types.

### ACTA2 missense variant disrupting R179 alters the nuclear localization of αSMA

To study whether the αSMA R179 variant alters nuclear localization, *Acta2*^−/−^ SMCs were infected with WT and mutant R179C *Acta2* expression constructs. R179C mutant αSMA localizes significantly less to the nucleus than WT αSMA without affecting the localization of β-actin (Fig. 3A, Supplemental Fig. VIA). Interestingly, *Acta2*^−/−^ SMCs expressing R179C αSMA also have decreased levels of calponin and SM22α when compared with cells expressing WT αSMA (Fig. 3A, Supplemental Fig. VIB). Co-immunoprecipitation with antibodies directed against either INO80 or BRG1 indicate decreased association of R179C αSMA with chromatin remodeling complexes compared with WT αSMA (Fig. 3B,C). By contrast, more β-actin is co-precipitated with INO80 in the cells expressing R179C mutant αSMA compared with WT (Fig. 3B). Finally, ChIP pulldowns with αSMA and β-actin antibodies confirm decreased binding of R179C mutant αSMA to the promoter regions of SMC-specific genes, while β-actin binding is increased in the cells expressing mutant compared with WT αSMA (Fig. 3D). There are no significant differences in binding of αSMA on the *Klf4* promoter, suggesting a specific function of αSMA on SMC-specific gene promoters. To further assess whether loss of nuclear αSMA has a functional impact, ChIP pulldowns were performed with antibodies against a histone marker of transcriptional activation, H3K4me3, and a marker of transcriptional repression, H3K27me3. The CArG box regions of SMC-specific genes have significantly decreased H3K4me3 in cells expressing R179C mutant αSMA compared with WT αSMA (Figure 3E). By contrast, there is no change in these histone modifications on the *Klf4* promoter. Taken together, these results suggest that arginine 179 alteration disrupts the nuclear localization and function of αSMA, and that this loss of nuclear function leads to changes in SMC-associated genomic loci.

### Mouse SMCs with knock-in R179C mutation are not fully differentiated and have decreased levels of nuclear αSMA

We generated a SMC-specific *Acta2* R179C knock-in mouse model (termed *Acta2*^SMC-R179C/+^) and validated that the mutation was present in 66% of SMCs in the aortic tissue *in vivo*, but SMCs explanted from the ascending aorta are a pure heterozygous population by RNA sequencing and 2D gel analyses.^15^ These findings suggest that the *Acta2*^SMC-R179C/+^ SMCs proliferate more rapidly than the WT SMCs, and we found that both proliferation and migration were increased in the mutant SMCs when compared to SMC explanted from WT mouse ascending aortas (Fig. 4A,B). *Acta2*^SMC-R179C/+^ SMCs also have significantly reduced expression and levels of contractile proteins, *Cnn1, Tagln*, and *Myh11* (Fig. 4C) and increased expression of pluripotency markers *Nanog, Klf4, Oct4*, and *Sox2* when compared with WT SMCs (Fig. 4D). Please note that interpretation of *Oct4* expression may be complicated by the existence of pseudogenes.^16^ Protein levels of calponin, SM22α, and SMMHC are decreased in *Acta2*^SMC-R179C/+^ SMCs consistent with the reduced RNA expression (Fig. 4E, Supplemental Fig. VIIA). *Acta2*^SMC-R179C/+^ SMCs have reduced levels of the myocardin related transcription factor A (Mk11), a cofactor that binds to semm response factor (SRF) to drive contractile gene expression (Fig. 4E, Supplemental Fig. VIIA). Since a highly migratory behavior is a hallmark of neural crest cell (NCC) progenitors,^17^ the less differentiated phenotype of *Acta2*^SMC-R179C/+^ SMCs may represent failure of NCCs to completely differentiate into SMCs during development rather than phenotypic modulation of differentiated SMCs.

*Acta2*^SMC-R179C/+^ SMCs have significantly decreased accumulation of αSMA in the nucleus and concomitant increased cytosolic accumulation. TGFβ1 treatment increases αSMA levels in both WT and *Acta2*^SMC-R179C/+^ SMCs (Fig. 4F, Supplemental Fig. VIIB). WT SMCs have nuclear αSMA and β-actin present with or without latrunculin A treatment, whereas β-actin in the nuclear fraction of *Acta2*^SMC-R179C/+^ SMCs decreases further with latrunculin A treatment (Fig. 4G, Supplemental Fig. VIIC). Immunostaining of αSMA in WT and mutant SMC nuclei followed by quantitation of fluorescence intensity confirms reduced nuclear αSMA in *Acta2*^SMC-R179C/+^ SMCs (Fig. 4H,I). To determine if association of αSMA with chromatin remodeling complexes is altered in *Acta2*^SMC-R179C/+^ SMCs, co-immunoprecipitation with antibodies directed against either INO80 or BRG1 were pursued. Co-precipitation of αSMA with chromatin remodeling complexes was decreased in *Acta2*^SMC-R179C/+^ SMCs, as was co-precipitation of β-actin, although TGFβ1 treatment rescues actin interactions with these complexes (Fig. 4J, Supplemental Fig. VIID).

The R179C mutation causes significant disruption of polymerization of αSMA in addition to the loss of nuclear function described here.^18^ To confirm that the decreased differentiation of *Acta2*^SMC-R179C/+^ SMCs is not due to cytosolic actin disruptions, WT mouse SMCs were treated with an αSMA disrupting peptide (SMAfp) or with a peptide designed to disrupt skeletal α-actin (SKAfp) as a control. These peptides have been previously characterized,^19^ and we previously showed that treatment with SMAfp marginally increases SMC proliferation through increased expression of PDGFRβ.^20^ Here, cells were treated with 5 μg/mL SMAfp to completely disrupt αSMA filaments, while SKAfp moderately affects αSMA filament formation (Fig. 4K). Treatment with 1 μg/mL SMAfp partially disrupts αSMA filaments, with no disruption by 1 μg/mL SKAfp (Supplemental Fig. IXA). Neither SMAfp nor SKAfp treatment at either dose affected nuclear localization of αSMA or the expression or levels of SMC contractile markers (Fig. 4L, Supplemental Fig. VIII, IX). Thus, SMCs with disrupted SMA polymerization do not de-differentiate, supporting that the lack of differentiation in *Acta2*^SMC-R179C/+^ SMCs is not due to disruption of αSMA polymerization.

### Mouse SMCs with knock-in R179C mutation have altered chromatin accessibility

To determine if decreased nuclear actin in *Acta2*^SMC-R179C/+^ SMCs alters global chromatin remodeling, assay for transposase-accessible chromatic (ATAC)-sequencing was pursued in WT and *Acta2*^SMC-R179C/+^ SMCs. We identified 2466 peak regions with greater than 1.5-fold differential accessibility, including 1018 peaks with increased accessibility and 1448 peaks with decreased accessibility in *Acta2*^SMC-R179C/+^ SMCs. Integrated peak region-gene association calls and pathway analysis using GREAT were performed (Supplemental Fig. XA,B). GO term analysis of the genes associated with peaks of decreased accessibility in *Acta2*^SMC-R179C/+^ SMCs shows enrichment of multiple biological processes related to muscle and cardiac cell development and contraction, consistent with the lack of differentiation observed in these SMCs (Fig. 5A). In contrast, GO terms associated with regions of increased accessibility in *Acta2*^SMC-R179C/+^ SMCs include terms related to cortical actin cytoskeleton or actomyosin structure organization, which are terms associated with the cortical actin rearrangements necessary for cell migration (Fig. 5B). These differences in chromatin accessibility align with differences in gene expression and cellular behavior in *Acta2*^SMC-R179C/+^ SMCs, providing evidence that chromatin remodeling changes due to loss of nuclear actin in* Acta2*^SMC-R179C/+^ SMCs may underlie the lack of differentiation and maintenance of some NCC phenotypic features.

### Mouse SMCs with knock-in R179C mutation are less differentiated in vivo

*Acta2*^SMC-R179C/+^ mice are mosaic with knock-in of the R179C variant in approximately 67% of SMCs based on single cell RNA-sequencing (scRNA-seq) of aortic and carotid artery tissue from these mice.^15^ Transcriptomic data from WT and *Acta2*^SMC-R179C/+^ aortic cells visualized in UMAP space identified two distinct clusters of SMCs in *Acta2*^SMC-R179C/+^ mice, one of which overlapped with the single SMC cluster in WT mice (Fig. 5C). Based on analysis of the transcriptomic data, the “SMC1” cluster represents cells without the R179C variant and the “SMC2” cluster represents cells with the variant. Data from cells in the SMC clusters from WT and *Acta2*^SMC-R179C/+^ tissue were assessed for differentially expressed genes (DEGs), and 289 DEGs were identified, with 122 genes downregulated and 167 genes upregulated in SMC2 cluster when compared with WT SMCs and SMC1 clusters (Fig. 5D). GO term enrichment analysis identified 10 terms significantly upregulated in SMC2, including regulation of cell proliferation (Fig. 5E). To visualize these changes in cell proliferation, we combined all genes represented in GO term 0008283 (Cell population proliferation) and visualized the pooled expression of these genes in the SMC clusters in UMAP space as well as quantified their expression in SMC1 vs. SMC2 cells to confirm significant enhancement in SMC2 cells (Fig. 5F). To assess whether SMC2 cells are less differentiated, we examined SMC marker gene expression in SMC1 compared with SMC2 and found significantly decreased expression of *Myh11, Cnn1*, and *Actg2* (Fig. 5G), with *Actg2* being the most significantly downregulated gene in the SMC2 cluster. However, *Acta2* expression was significantly upregulated in SMC2 cells, consistent with what we observed in *Acta2*^SMC-R179C/+^ SMCs *in vitro* (Fig. 5G).

To globally link chromatin accessibility changes with transcriptomic changes, gene lists from the *in vitro* ATAC-seq dataset and the *in vivo* scRNA-seq dataset were compared. Genes appearing in both lists were graphed in two dimensions according to differential expression and differential accessibility (Supplemental Fig. XC). Genes with both increased expression and accessibility include *Klf4*, which encodes a pluripotency and SMC-plasticity associated transcription factor (Supplemental Fig. XIA)^21^ and *Smad7*, which encodes an inhibitory Smad that blocks canonical TGFβ1 signaling (Supplemental Fig. XIB).^22^ Knockdown of *Smad7* expression by shRNA increases expression of SMC contractile genes (Supplemental Fig. XIC), supporting that this gene plays a role in regulating SMC differentiation. Genes with both decreased expression and accessibility are *Itgb1* and *Synpo2*, both of which have a CArG box element in their promoters and are highly expressed in differentiated SMCs (Supplemental Fig. XIIA,B).^23, 24^ GO term enrichment analysis performed on genes transcriptionally upregulated and associated with increased chromatin accessibility in *Acta2*^SMC-R179C/+^ SMCs identified terms associated with proliferation and migration of cells (Fig. 5H). Similar analysis on genes transcriptionally downregulated and with decreased chromatin accessibility identified two terms: “muscle cell differentiation” and “muscle cell development” (Fig. 5H). These results indicate that altered chromatin accessibility drives the transcriptional changes in *Acta2*^SMC-R179C/+^ SMCs, leading to decreased differentiation and increased migration and proliferation.

### *Patient iPSC-derived* ACTA2 *p.R179C SMCs lack nuclear αSMA and are less differentiated*

To confirm that human SMCs with *ACTA2* p.R179 missense variants are similar to our mouse model, fibroblasts from patients with *ACTA2* R179 altered to either cysteine or histidine were reprogrammed into iPSCs and differentiated into SMCs via NEPC intermediates (n=3 patient, n=3 control; demographic information in Supplemental Fig. XIIIA). The patient-derived SMCs are less differentiated relative to controls, as illustrated by decreased levels of contractile proteins (Fig. 6A, Supplemental Fig. XIIIB-D), decreased expression of SMC contractile genes (Fig. 6B), and increased retention of pluripotency gene expression (Fig. 6C). Patient-derived *ACTA2* p.R179C SMCs have significantly lower accumulation of αSMA in the nucleus at baseline. Levels of αSMA increase in the cytosol and nucleus with TGFβ1 exposure in the mutant SMCs, but the ratio of nuclear to cytosolic αSMA does not change (Fig. 6D, Supplemental Fig. XIVA-C). Nuclear αSMA levels are also significantly reduced in *ACTA2* p.R179 cells at the NEPC stage, supporting that loss of nuclear αSMA during development could be the cause of incomplete differentiation of these cells (Fig. 6E, Supplemental Fig. XIVD). The *ACTA2* p.R179C patient-derived SMCs show significant reduction in the association of both αSMA and β-actin to the INO80 and BAF ATP-dependent chromatin remodeling complexes when compared with control SMCs (Fig. 6F, Supplemental Fig. XIVE).

R179H αSMA has defects in polymerization in *in vitro* studies.^18^ Confirming this, *ACTA2* p.R179C SMCs have decreased αSMA filament formation (Supplemental Fig. XV). The transcriptional coactivator MKF1 binds to monomeric actin in the cytosol, thus decreasing its binding to SRF in the nucleus and reducing SRF-driven expression of SMC differentiation markers.^14^ To determine whether this pathway contributes to de-differentiation of *ACTA2* p.R179 cells, actin polymerization was assessed by an ultracentrifugation-based F/G actin assay. Cells derived from an SMDS patient had no change in F to G actin ratio compared with control cells (Fig. 6G). *ACTA2* p.R179C SMCs have a significant increase in the ratio of nuclear to cytosolic MKL1 compared with controls (Fig. 6H,I). These results suggest that de-differentiation of the mutant cells is not the result of the MKL1/SRF axis.

To accurately assess whether loss of nuclear αSMA prevents complete differentiation of SMCs, Crispr/Cas9 genomic editing was used to induce a homozygous loss-of-function allele in *ACTA2* in control iPSCs. These cells were differentiated into NEPCs and then SMCs, and the cells with near-total loss of *ACTA2* expression also had decreased expression of other SMC contractile genes (Fig. 6J) and increased expression of pluripotency-associated genes (Fig. 6K) comparable to cells derived from *ACTA2* p.R 179 patients. These results further support that loss of nuclear αSMA prevents the complete differentiation of SMCs.

### *Single cell transcriptomics of the aorta of patient with* ACTA2 *p.R179H confirms dedifferentiation in vivo*

To determine the functional consequences of *ACTA2* p.R179 variants in human aortic disease *in vivo*, the ascending aorta from an 8-year-old child with SMDS due to a *de novo ACTA2* c.536G>A variant (p.R179H) was assessed using single cell transcriptomics, along with a 12mm diameter distal ascending aortic tissue sample from a 2-year-old heart donor as a control (Fig. 7A,B). The patient had classic features of SMDS including congenital mydriasis, intestinal malrotation, repair of a patent ductus arteriosus at two weeks of age, and progressive aneurysmal enlargement of the root and ascending aorta (37mm diameter, Z-score 11.0 at the time of surgery).^4^ We performed enzymatic digestion and single-cell RNA sequencing (scRNA-seq) on fresh aneurysm tissue at the time of elective aortic repair surgery using a validated protocol.^25, 26^ Following single cell captures and mRNA library preparation, samples were sequenced and integrated into a joint dataset (6,263 cells) using standard workflows within the Seurat package in R (Fig. 7C).^27, 28^ Low-resolution clustering of the integrated dataset identified 10 cell types, including SMC, fibroblast, endothelial (EC), and macrophage clusters (Fig. 7D), as well as small populations unique to the *ACTA2* p.R179H sample including mast cells (enriched *TPSB2, CPA3, KIT* expression) and chondromyocytes (*COL2A1, ACAN*, and *SOX9*-expressing, Supplemental Fig. XVI). To simulate a ‘pseudobulk’ transcriptomic comparison of all resident aortic SMCs of the *ACTA2* p.R179H and control, we sub-selected the SMC cluster and performed differential expression testing (Fig. 7E). Consistent with *in vitro* findings in *ACTA2* p.R179 iPSC-derived SMCs, we identified significantly decreased expression of SMC markers (*CNN1* and *MYH11*) while also identifying increased *ACTA2* expression (Fig. 7F). Globally, 812 DEGs between the case and control were identified in the SMC clusters. To broadly characterize the phenotypic consequences of decreased SMC dedifferentiation in the patient’s SMCs, we performed gene set enrichment analysis (GSEA) on this gene list ranked by fold change, and identified 30 statistically significant (FDR < 0.05) gene ontology (GO) pathways enriched in *ACTA2* p.R179H SMC cluster including multiple immunomodulatory pathways, biological adhesion, secretion, chemotaxis, and cartilage development (Fig. 7G). *ACTA2* p.R179H SMCs occupy a broader distribution in uniform manifold approximation and projection (UMAP) space that includes several distinct projections, suggesting multiple, heterogeneous alternate cell fates for poorly differentiated *ACTA2* p.R179H SMCs. To examine this, we first generated a SMC contractile score comprising composite expression of core contractile genes (*MYH11, CNN1, MYL9, ACTA2*) to establish the heterogeneity of SMC maturity in UMAP space within the dataset (Fig. 7H). Progressively reduced expression of these markers correlates with multiple distinct subsets within the broader SMC dataset populated almost entirely by *ACTA2* p.R179H cells. Although expression of some stem cell markers was not present in these subsets (e.g., *OCT4*, *SOX2*), there are uniquely activated markers in these phenotypic offshoots. One subset expresses both epithelial-like markers (*KRT1*7 and other cytokeratins) and ‘typical’ ECM synthetic markers (*FN1, FBLN2* and *CXCL12*), while a distinct subset is distinguished by markers associated with neural progenitor cells including *GDF10*, WNT signaling modulators (e.g. *FRZB* and *SOST*), neurogranin (*NRGN*), and *IGFBP5* (Fig. 7I). All five of these genes are targets of EZH2 in the ENCODE database; EZH2 is a component of the polycomb repressive complex and is required for neural crest cell-derived cartilage and bone formation.^29^ The interpretation of these results is limited by the availability of only one tissue sample from an *ACTA2* p.R179 patient and by the imperfect matching of the control and patient tissue samples. Nonetheless, these results indicate that the *ACTA2* p.R179 alteration leads to poorly differentiated aortic SMCs *in vivo*, and suggests that the consequence of this loss of differentiation is increased plasticity and diverse pathologic alternative cell fates possibly resulting from disease-associated stimuli *in vivo*.

## Discussion

The data presented here demonstrate a unique role for αSMA in the nucleus that is dismpted when arginine 179 in αSMA is altered. Specifically, αSMA is present in the nucleus in NEPC cells and nuclear levels increase with SMC differentiation. Nuclear αSMA co-precipitates with the INO80 and BAF chromatin remodeling complexes and associates with CArG box elements in the promoters of SMC differentiation genes, and altering R179 in αSMA disrupts these nuclear associations. Reduction of nuclear αSMA in *Acta2*^SMC-R179C/+^ SMCs is associated with loss of differentiation and augmented proliferation and migration when compared to WT SMCs. Importantly, decreased differentiation of *Acta2*^SMC-R179C/+^ SMCs is not driven by depolymerized actin monomers in the cytosol, as MKL1 is localized to the nucleus in mutant SMCs and disruption of actin polymerization in WT SMCs does not decrease differentiation. Similarly, a lack of SMC differentiation is observed *in vivo* in the mutant SMC clusters in the inducible knock-in *Acta2*^SMC-R179C/+^ mouse model, although we have not confirmed lack of nuclear αSMA localization *in vivo*. Importantly, *Acta2*^SMC-R179C/+^ mice do not develop aortic disease,^15^ so alterations in SMC phenotype *in vivo* are not secondary to aortic disease progression. In αSMA R179 mutant SMCs, alterations in chromatin accessibility correlate with gene expression changes, and therefore potentially underlie the lack of differentiation and increased proliferation and migration, i.e., phenotypes associated with stem cells. Knockout of *ACTA2* using Crispr/Cas9 editing of iPSCs similarly disrupted the differentiation of NEPCs to SMCs. Taken together, these data support a model that nuclear αSMA is required during SMC differentiation to facilitate chromatin remodeling changes that drive the transition from a progenitor cell to a fully differentiated SMC. Altering R179 in αSMA disrupts the nuclear localization and function of αSMA and prevents the full differentiation of a progenitor cell to a SMC, resulting in SMCs that maintain stem cell features like increased migration and proliferation.

We hypothesize that decreased levels of αSMA in the nucleus underlie the lack of differentiation in heterozygous *ACTA2* R179 SMCs. Treatment with TGFβ1 increases αSMA nuclear localization in the R179 mutant cells and partially rescues the decreased differentiation. However, the rescue of SMC contractile protein levels is incomplete, and *ACTA2* p.R179 cells undergo longer-term treatment with TGFβ1 during NEPC to SMC differentiation and still fail to fully differentiate. These data suggest the possibility that R179 mutant αSMA has functional defects in the nucleus even when nuclear levels are increased by TGFβ1 treatment, and future studies will address this possibility.

Based on single cell transcriptomics of an *ACTA2* p.R179H patient’s aortic tissue, the hypothesis that the mutation disrupts differentiation of NCCs to SMCs is supported by the increased phenotypic plasticity of SMCs, which have multiple trajectories of cell modulation. Consequently, *ACTA2* p.R179H patient cells have unique phenotypic trajectories with increased expression of markers of other NCC-derived cell types, including keratinocytes and neuronal progenitors.^30^ Notably, a cluster of chondromyocyte-like cells appears in the *ACTA2* p.R179H aortic tissue but not the control aortic tissue, and although this cluster is not confirmed to be SMC-derived, NCCs can also differentiate into chondrocytes.^31^ Importantly, the *ACTA2* p.R179H SMC cluster shares some typical dedifferentiated SMC transcriptional profiles present in single cell transcriptomic data from a patient with Marfan syndrome (e.g. *FN1, COL1A1*), but also expresses multiple markers not found in the Marfan patient SMC cluster (Supplemental Fig. XVII).^25, 32^ The *ACTA2* p.R179H SMCs also do not show activation of TGFβ1-driven signaling in contrast to the Marfan patient SMCs (Supplemental Fig. XVIIF). Single cell transcriptomic data from atherosclerotic plaques shows modulated SMCs that are dedifferentiated with increased chondrogenic gene expression, a similar transcriptional profile to the chondromyocyte cluster in the *ACTA2* p.R179H aortic tissue, supporting the conclusion that these cells are also SMC-derived.^33^ Interestingly, we found that multiple gene targets of EZH2 are upregulated in the *ACTA2* p.R179H tissue. A recent paper showed in the absence of β-actin, BRG1 genomic association is globally depleted, leading to increased EZH2 recruitment, and in this context EZH2 acts as a transcriptional activator of a subset of genes.^34^ EZH2 in the cytosol has also been shown to regulate actin polymerization,^35^ and it has been speculated that nuclear EZH2 could similarly regulate nucleoskeletal assembly of actin filaments.^36^ EZH2 is a methyltransferase responsible for trimethylation of H3K27.^36^ H3K27me3 mark at the *Tagln* and *Cnn1* loci is increased in SMCs expressing R179C αSMA, suggesting there may be increased EZH2 activity in cultured cells as well as the aortic tissue.

The molecular alterations in SMCs with heterozygous alterations at R179 in αSMA, specifically increased proliferation and migration, potentially contribute to the early childhood onset occlusive lesions in arteries in SMDS patients based on the fact that these lesions are characterized by intimal cells that occlude the lumen and stain positive for SMC markers.^8^ The occlusive vascular disease in SMDS patients is phenotypically similar to patients with Grange Syndrome, another condition characterized by childhood onset moyamoya-like cerebrovascular disease that results from homozygous loss-of-function mutations in *YY1AP1*.^37^ We determined that YY1AP1 associates with the INO80 complex and αSMA in the nucleus of SMCs; thus loss of YY1AP1 could result in similar SMC phenotypic changes as the *ACTA2* R179 alteration. Furthermore, mice with global deficiency in *Brg1*, which encodes the ATPase component of the BAF complex, have decreased expression of SMC contractile genes, and loss of BRG1 prevents myocardin from activating expression of SMC contractile genes in cultured SW13 cells.^38, 39^ We determined that αSMA co-precipitates with these two ATP-dependent chromatin remodeling complexes, the INO80 complex and the BAF complex (also called SWI/SNF). In the INO80 complex, β-actin is part of a module containing Arp4 and Arp8, and this module acts as a sensor for the complex to bind linker DNA and triggers a conformational switch to enable nucleosome binding.^40–42^ In the BAF complex, the actin-Arp module couples the motions of the ATPase module and the base module to regulate complex functions.^43^ Loss or mutation of β-actin has previously been shown to impact the functional activity *in vitro* of both the INO80 complex and the BAF complex.^34, 44^ Future studies will determine if αSMA assumes the function of β-actin in the complex, and if αSMA alters the structure, function, or targeting of these chromatin remodeling complexes in a manner unique from β-actin. The phenotypic overlap of the SMDS and Grange syndrome patients, along with the data presented here and loss of differentiation in mouse models with deficiency of BRG1, leads us to hypothesize that INO80 and BAF chromatin remodeling complex function is critical for SMC fate determination. Furthermore, incomplete SMC differentiation due to chromatin remodeling disruptions leads to a progenitor cell-like phenotype of increased proliferation and migration that potentially contributes to the occlusive cerebrovascular disease in SMDS patients. SMDS patients overwhelmingly have pathogenic variants affecting *ACTA2* R179, but a recent case was reported of a patient with SMDS and a miR-145-5p pathogenic variant.^45^ miR-145 represses expression of pluripotency markers like *OCT4* and *SOX2* to control smooth muscle cell fate.^46, 47^ This report therefore aligns with our hypothesis that incomplete differentiation of SMCs and retention of progenitor phenotypes underlies SMDS pathogenesis.

Previous data addressing the role of nuclear β-actin in cellular differentiation support that efflux of nuclear β-actin during development is a mechanism of cell fate transition. Importantly, we observed decreased nuclear β-actin in NEPCs relative to differentiated SMCs (Fig. 1G). In *Xenopus* oocytes, high levels of nuclear β-actin are present due to low expression of its nuclear exporter, Xpo6. As oocytes differentiate, Xpo6 expression is increased, β-actin is exported from the nucleus, and differentiation is initiated.^48^ In epidermal progenitors, mechanical signaling reduces nuclear β-actin levels, which in turn promotes repressive histone modification-induced gene silencing that blocks lineage commitment.^49^ Additionally, β-actin deficiency in mouse embryonic fibroblasts (MEFs) found that nuclear β-actin directly modulates cell fate. Loss of β-actin prevents MEFs from reprogramming into neuronal cell types and is associated with increased accumulation of repressive histone modifications, and these defects can be partially rescued by expression of NLS-tagged β-actin.^10,11^ β-actin knockout MEFs have compensatory upregulation of αSMA, and importantly, an ATAC-seq analysis comparing open chromatin regions in β-actin deficient and WT cells showed repression of neuronal differentiation genes.^34^ One of the top upregulated pathways in the gene ontology analysis of the ATAC-seq data was “vascular development”, which potentially supports our hypothesis that nuclear αSMA preferentially activates genes critical for SMC differentiation over β-actin. Our own ATAC-seq analysis further supports our hypothesis by identifying that, in *Acta2*^SMC-R179C/+^ SMCs with less nuclear αSMA, genes associated with cortical actin rearrangements needed for cellular migration are more accessible and genes associated with muscle differentiation and contraction are less accessible. In summary, these studies all indicate that nuclear actin is an epigenetic remodeling factor that helps coordinate chromatin accessibility and gene expression and establish cell fate, and our data support that αSMA, rather than β-actin, coordinates these activities in the nucleus of developing SMCs.

β-actin has been shown to play multiple and increasingly complex roles in regulation of nuclear functions from transcription to DNA repair to chromatin remodeling.^50^ Nuclear β-actin directly regulates global transcription by functionally interacting with RNA polymerase II.^51^ Relevant to SMC specific gene transcription, nuclear β-actin has been shown to regulate activity of SRF through binding and sequestration of MKL1 similar to the function of G-actin in the cytosol.^52, 53^ Impairment of nuclear localization of β-actin by knockdown of its nuclear importer Ipo9 or of actin polymerization in the nucleus using drugs like latrunculin A hinders DNA damage response.^54, 55^ Besides its roles in regulating chromatin structure through ATP-dependent chromatin remodeling complexes, nuclear β-actin may also be involved in compartment level regulation of 3D genomic architecture, in particular by regulating the distribution of heterochromatin marked by HP1.^50^ Future studies will focus on which of these important nuclear functions αSMA shares with β-actin, and on whether αSMA versus β-actin involvement differentially affects these functions. Preliminarily, we showed that both αSMA and β-actin co-precipitate with chromatin remodeling complexes and are strongly negatively correlated with HP1, a marker of heterochromatin, by colocalization analysis, but this study is only the beginning of defining αSMA nuclear functions.

The mechanism preventing nuclear localization of R179 mutant αSMA is unidentified. In *Acta2*^SMC-R179C/+^ SMCs, there is a greater than 50% reduction in nuclear αSMA in heterozygous cells, indicating a dominant negative effect of mutant αSMA on WT αSMA nuclear localization. We have previously reported that *ACTA2* pathogenic variants disrupting arginine 258 also predispose to moyamoya-like cerebrovascular disease,^1^ and found that methylation at the corresponding amino acid in yeast actin or human β-actin (R256) is required for nuclear function.^12^ Arginine methylation is a post-translational modification linked with nuclear functions of proteins, including chromatin organization, gene expression, and RNA processing.^56^ Methylation of yeast actin at R177 does occur;^12^ and, in the context of missense mutations affecting R179, loss of methylation would potentially dismpt nuclear function of the protein. Interestingly, a second band for nuclear αSMA migrating at a slightly higher molecular weight was noted on immunoblots in control NEPCs (Fig. 1G), raising the possibility that additional post-translational modifications of αSMA could be important for its nuclear function. Alternatively, R179 mutant αSMA could be excluded from the nucleus due to aberrant protein interactions, including altered interactions with proteins responsible for importing and exporting αSMA to the nucleus. β-actin binds to cofilin and is then imported by Ipo9, while profilin-bound actin is exported by Xpo6.^54, 57^ *In vitro* studies previously showed that both R179 and R258 mutant αSMA bind more tightly to profilin,^18, 58^ and we found less nuclear profilin in cells expressing R179 mutant αSMA compared with WT (Fig. 3A and data not shown). Future studies will focus on profilin or additional binding proteins to explore the mechanism excluding R179 mutant αSMA from the nucleus.

This work establishes a novel and critical role for αSMA as an epigenetic factor involved in the developmental specification of SMCs. We further hypothesize that cardiac and skeletal muscle-specific α-actins play a similar role by accumulating in the nucleus during development and guiding myocyte-specific chromatin remodeling and fate specification. Loss of nuclear αSMA with *ACTA2* R179 mutation causes alterations in chromatin accessibility, leading to incomplete differentiation of SMCs. The consequence of this incomplete differentiation is retention of stem cell-like phenotypes of increased proliferation and migration, which may contribute to the occlusive vascular disease in SMDS and multiple trajectories of SMC modulation.

## Materials and Methods

Detailed descriptions of all materials and methods for these studies is included in the Supplemental Materials.

## Figures and Tables

**Figure 1.** αSMA localizes to the nucleus concurrently with SMC differentiation. A) Immunoblot of fractionated protein lysates from WT mouse explanted SMCs shows αSMA localizes to the nucleus in SMCs, and both cytosolic and nuclear αSMA levels increase with TGFβ1 stimulation, while PDGF-BB stimulation does not affect nuclear accumulation of αSMA. B) 2D gel electrophoresis shows both αSMA and β-actin in the nucleus of SMCs, with significant enrichment of αSMA over β-actin in the nucleus with TGFβ1 stimulation. C) LatmnculinA (LtA) treatment does not alter the ratio of nuclear to cytosolic αSMA on immunoblot. D-F) Immunostaining of isolated nuclei (D) shows increased nuclear αSMA after treatment with TGF㬡 or PDGF-BB, quantified in (E) and confirms absence of αSMA signal in areas of heterochromatin by colocalization analysis between HP1 (red) and αSMA (green), quantified in (F). G) Immunoblot of fractionated protein lysates taken at timepoints during the differentiation of NEPCs (day 0) to SMCs (day 12) shows early and dramatic accumulation of nuclear αSMA. β-actin is decreased in the nucleus of NEPCs, and other SMC proteins increase during differentiation as expected. H) Quantitative RT-PCR shows exponential increases of SMC contractile gene expression during the timecourse of NEPC to SMC differentiation. Timepoints match between G and H. Data shown are representative of at least three independent experiments. Quantitations of immunoblots can be found in Supplemental Figure I. Negative controls for immuno staining can be found in Supplemental Figure IIA. *p<0.05, ****p<0.0001

**Figure 2.** αSMA binds chromatin remodeling complexes and the promoters of SMC contractile genes. A) Co-immunoprecipitation pulldowns of nuclear protein lysates from SMCs with an antibody directed against αSMA show interactions with several members of the INO80 and BAF chromatin remodeling complexes. The dotted line separates two independent pulldown experiments. B) Reciprocal pulldowns with an antibody directed against INO80, a subunit of the INO80 complex, confirm both αSMA and β-actin associate with the complex in the nucleus of SMCs. C) Reciprocal pulldowns with an antibody directed against BRG1, a subunit of the BAF complex, confirm both αSMA and β-actin associate with the complex in the nucleus of SMCs. Negative control pulldowns using species-matched IgG were performed for all experiments and are shown in the figure. D) Chromatin immunoprecipitation (ChIP) pulldowns of crosslinked SMC lysates shows that αSMA binds to the CArG box region of SMC contractile gene promoters, and this binding is increased after TGFβ1 stimulation. By contrast, no significant changes are found at the *Actb* promoter. E) Sequential pulldown of αSMA followed by INO80 or the reciprocal INO80 followed by αSMA reveals co-enrichment of these two proteins on CArG box regions of SMC contractile genes. Single pulldown controls can be found in Supplemental Figure III. F) Lentiviral-induced overexpression of empty vector or β-actin or αSMA tagged with a nuclear localization sequence (EV, β-NLS, and α-NLS labels, respectively) shows that only α-NLS increases accumulation of contractile proteins in WT mouse SMCs. Immunoblot quantitations are in Supplemental Figure IV. G) Collagen gel contraction assay shows that α-NLS-infected cells are more contractile than cells with EV or β-NLS. Data shown are representative of at least three independent experiments. *p<0.05, **p<0.01, ***p<0.001, ****p<0.0001

**Figure 3.** R179C mutation impairs nuclear αSMA localization and function when expressed in *Acta2* KO SMCs. A) Lentiviral infection with a construct overexpressing either WT αSMA or R179C mutant αSMA shows decreased nuclear localization of the mutant αSMA. Levels of SMC contractile proteins are also moderately decreased. B, C) Co-immunoprecipitations of nuclear protein lysates with an INO80 antibody (B) or a BRG1 antibody (C) show decreased association of R179C mutant αSMA with the chromatin remodeling complexes. Negative control pulldowns using species-matched IgG were performed for all experiments and are shown in the figure. D) Chromatin immunoprecipitation (ChIP) pulldowns of crosslinked SMC lysates shows that R179C mutant αSMA binds significantly less to the CArG box regions of SMC contractile genes. E) Chromatin immunoprecipitation (ChIP) pulldowns of crosslinked SMC lysates shows that cells expressing R179C mutant αSMA have decreased H3K4me3 on the CArG box regions of SMC contractile genes. Data shown are representative of at least three independent experiments. Quantitations of immunoblots can be found in Supplemental Figure V. *p<0.05, **p<0.01, ***p<0.001, ****p<0.0001

**Figure 4.** Heterozygous inducible knock-in of the R179C mutation in mouse SMCs leads to loss of nuclear αSMA and decreased differentiation of SMCs. A) *Acta2*^SMC-R179C/+^ SMCs proliferate more rapidly than controls at baseline as assessed by BrdU incorporation ELISA. B) *Acta2*^SMC-R179C/+^ SMCs migrate more rapidly than controls as assessed by Transwell assay without chemoattraction. C,D) Quantitative RT-PCR shows significantly decreased expression of SMC contractile genes (C) and significantly increased expression of pluripotency-associated genes (D) in *Acta2*^SMC-R179C/+^ SMCs compared with WT. E) Immunoblot analysis confirms decreased accumulation of SMC contractile proteins in *Acta2*^SMC-R179C/+^ SMCs compared with WT, including decreased accumulation of the transcription factor Mkll. F) Immunoblot analysis of fractionated lysates confirms decreased nuclear accumulation of both αSMA and β-actin in *Acta2*^SMC-R179C/+^ SMCs compared with WT. G) Latmnculin (LtA) treatment further decreases nuclear accumulation of both αSMA and β-actin in *Acta2*^SMC-R179C/+^ SMCs, suggesting that contamination of the nuclear fraction may contribute to the positive nuclear signal in the mutant cells but not the WT. H) Immunofluorescent imaging of isolated nuclei confirms decreased nuclear localization of αSMA staining in *Acta2*^SMC-R179C/+^ SMCs. I) Quantitation of standard confocal microscopy images confirms decreased fluorescence intensity in the nuclei of *Acta2*^SMC-R179C/+^ SMCs. J) Co-immunoprecipitation with INO80 antibody confirms decreased association of both αSMA and β-actin with the INO80 chromatin remodeling complex in *Acta2*^SMC-R179C/+^ SMCs that is partially rescued by TGfβ1 treatment. Negative control pulldowns using species-matched IgG are shown in the figure. K) Treatment with 5 μg/mL SMAfp peptide completely disrupts cytosolic actin filaments visualized by immunofluorescent staining (αSMA green, phalloidin red, Dapi blue). L) Treatment with 5 μg/mL SMAfp peptide does not affect nuclear accumulation of αSMA and does not decrease levels of SMC contractile proteins. For K and L, SKAfp was used as a control. Data shown are representative of at least three independent experiments. Quantitations of immunoblots can be found in Supplemental Figure VII and VIII. *p<0.05, **p<0.01, ***p<0.001, ****p<0.0001.

**Figure 5.** *Acta2*^SMC-R179C/+^ mouse SMCs have altered chromatin accessibility *in vitro* and are hypodifferentiated *in vivo.* A) GO term analysis shows enrichment of genes associated with peaks of decreased chromatin accessibility in *Acta2*^SMC-R179C/+^ SMCs includes multiple terms related to muscle development and contraction. B) GO term analysis shows enrichment of genes associated with peaks of increased chromatin accessibility in *Acta2*^SMC-R179C/+^ SMCs includes multiple terms related to cortical actin cytoskeleton and actomyosin structure organization. C) Low-resolution cell clustering of compiled dataset from integrated single cell RNA-sequencing identifies 9 distinct cell types in WT and *Acta2*^SMC-R179C/+^+ mouse tissue. Prior analysis indicates mosaicism in *Acta2*^SMC-R179C/+^ mice with SMC2 cells harboring the R179C variant and SMC1 cells lacking the variant. D) Volcano plot of differentially expressed genes comparing SMC1 cluster vs. SMC2 cluster shows 289 differentially expressed genes. E) GO term enrichment analysis shows no terms enriched in SMC1 cluster and 10 terms including cell population proliferation enriched in SMC2 cluster. F) Composite proliferative score defined by composite expression of all genes from GO:0008283 (cell population proliferation) visualized in UMAP space highlights increased expression in SMC2, quantified in the violin plot. G) Violin plots for typical vascular smooth muscle cell markers depicting distribution of expression values for denoted genes within all SMCs in the dataset. H) GO term enrichment analysis on combined datasets of differentially expressed genes from scRNA-seq with differentially accessible regions from ATAC-seq shows increased accessibility and expression associated with migration and proliferation and decreased accessibility and expression associated with muscle cell differentiation and development.

**Figure 6.** Heterozygous patient-derived *ACTA2* p.R179C SMCs are less differentiated with reduced nuclear αSMA. A) Immunoblot analysis confirms decreased accumulation of SMC contractile proteins in *ACTA2* p.R179C iPSC-derived SMCs compared with control cells. B,C) Quantitative RT-PCR shows significantly decreased expression of SMC contractile genes (B) and significantly increased expression of pluripotency-associated genes (C) in *ACTA2* p.R179C iPSC-derived SMCs compared with control cells. D,E) Immunoblot analysis of fractionated lysates confirms decreased nuclear accumulation of both αSMA and β-actin in *ACTA2* p.R179C iPSC-derived SMCs (D) and NEPCs (E) compared with control cells. F) Co-immunoprecipitation with INO80 antibody confirms decreased association of both αSMA and β-actin with the INO80 chromatin remodeling complex in *ACTA2* p.R179C SMCs. Negative control pulldowns using species-matched IgG are shown in the figure. G) Ultracentrifugation-based F/G actin assay confirms no increased pools of actin monomers in *ACTA2* p.R179C SMCs. H) Immunofluorescent staining with an antibody against MKL1 (green) shows increased nuclear localization of the transcription factor in *ACTA2* p.R179C SMCs compared with controls, quantified in (I). J,K) SMCs differentiated from iPSCs subjected to Crispr/Cas9-induced knockout of *ACTA2* show decreased expression of contractile genes (J) and increased expression of pluripotency genes (K). Data shown are representative of at least three independent experiments. Quantitations of immunoblots and data from additional patient lines can be found in Supplemental Figures XIII and XIV. *p<0.05, **p<0.01, ***p<0.001, ****p<0.0001.

**Figure 7.** Single cell RNA sequencing of *ACTA2* p.R179H patient tissue confirms hypodifferentiated phenotype and increased plasticity *in vivo.* A) Three-dimensional CTA reconstruction and gross tissue specimen for ascending aortic aneurysm in 8-year old *ACTA2* p.R179H SMDS patient at time of operative repair. B) Gross tissue specimen of distal ascending aorta from healthy organ donor control. C) Integrated single cell RNA sequencing (scRNAseq) dataset from *ACTA2* p.R179H and donor control samples. D) Low-resolution cell clustering of compiled dataset identifies 10 distinct cell types. Dashed line highlights distinct UMAP projections of smooth muscle cell (SMC) subset selected for further analysis, red and black arrowheads denote disease-specific mast cell and chondromyocyte cell types, respectively. E) Overlaid UMAP projection of SMC partition demonstrating disease-specific distribution (blue). F) Violin plots for typical vascular smooth muscle cell markers depicting distribution of expression values for denoted genes within all SMCs in the dataset. G) Top 20 pathways enriched in *ACTA2* p.R179H SMCs by gene set enrichment analysis (GSEA) using differentially expressed genes ranked by fold change between genotypes. H) Composite SMC contractile score defined by composite expression of core mature SMC markers *CNN1, MYH11, MYL9,* and *ACTA2* in UMAP space highlights heterogeneous gene expression within the dataset and multiple distinct projections with reduced mature SMC gene profile. I) Feature plots depicting expression of mature SMC markers (*CNN1/MYH11*) and empirically determined markers for multiple alternate cell fate projections in UMAP space.

## Data Availability

Single cell RNA-sequencing datasets generated for this manuscript have been deposited in GEO and are available with accession number GSE201091. No novel code or algorithm was generated for this study. All reagents and resources applicable to this study are available from the corresponding authors upon reasonable request.
